# Supplementary material for: Magic cancellation point for vibration resilient ultrastable microwave signal synthesis
Source: Nat Commun. 2025 Aug 27;16:7997. doi: 10.1038/s41467-025-63369-3 (PMC12391395; doi:10.1038/s41467-025-63369-3)
Supplement: Supplementary file 1 — Supplementary Information [file 41467_2025_63369_MOESM1_ESM.pdf]

Supplementary Information: Magic Cancellation Point for Vibration Resilient Ultrastable Microwave Signal Synthesis

William Loh,[1,*] Dodd Gray,[1] Ryan Maxson,[1] Dave Kharas,[1] Jason Plant,[1]

Paul W. Juodawlkis,[1] Cheryl Sorace-Agaskar,[1] and Siva Yegnanarayanan[1]

[1]*MIT Lincoln Laboratory, Lexington, Massachusetts 02421, USA*

* Correspondence to william.loh@ll.mit.edu.

Supplementary Note 1: Stimulated Brillouin Scattering Laser Resonator

Supplementary Figure 1a shows a photograph of the fiber resonator resting in a copper enclosure having dimensions 3.1 inch $\times$ 3.5 inch $\times$ 1 inch. The enclosure is temperature controlled via a thermistor and thermoelectric cooler to a setpoint near room temperature. Extended Data Figure 1b plots the measured resonances of the slow and fast axis modes in the fiber SBS resonator. The linewidths are measured to be $\sim$110 kHz, yielding a loaded Q of 1.8×10^9. The corresponding PDH error signal is shown in Ext. Data Fig. 1c. Finally, Ext. Data Fig. 1d plots the measured photodetected amplitude after locking, which uses our single-photodetector scheme to demodulate both PDH error signals. Care is exercised to ensure the combined power of both pump lasers does not saturate the photodetector response. With the slow axis pump laser locked on resonance, the photodetected signal reduces to 60% of the total. When both the slow and fast axis pump lasers are locked, the signal amplitude reduces further to 25%.

Supplementary Note 2: Acceleration With Magic Cancellation Point

Supplementary Figure 2 depicts the effect of the magic cancellation point on the vibration sensitivity of the ODFD system. The input applied acceleration (Supplementary Fig. 2a) is first measured via an accelerometer mounted on the vibration platform. The applied acceleration consists of a sinusoidal modulation tone swept from 6 Hz to 100 Hz. The choice of a sinusoid, rather than broadband noise, is used for maximizing the applied vibration at a particular frequency under test. At each frequency, the voltage of the vibration platform is driven at the maximum voltage of 2 V before substantial nonlinearities appear. Supplementary Figures 2b and 2c show the resulting phase noise of the ODFD output in response to the applied vibration. In Supplementary Fig. 2b, the ODFD is operated with both SBS lasers locked to modes with the same polarization, and thus the system does not exhibit a magic cancellation point for vibration-induced fluctuations. In Supplementary Fig. 2c, when the SBS lasers are locked to orthogonal polarization modes at the magic cancellation point, the suppression in phase noise is observed to be >20 dB throughout the spectrum.

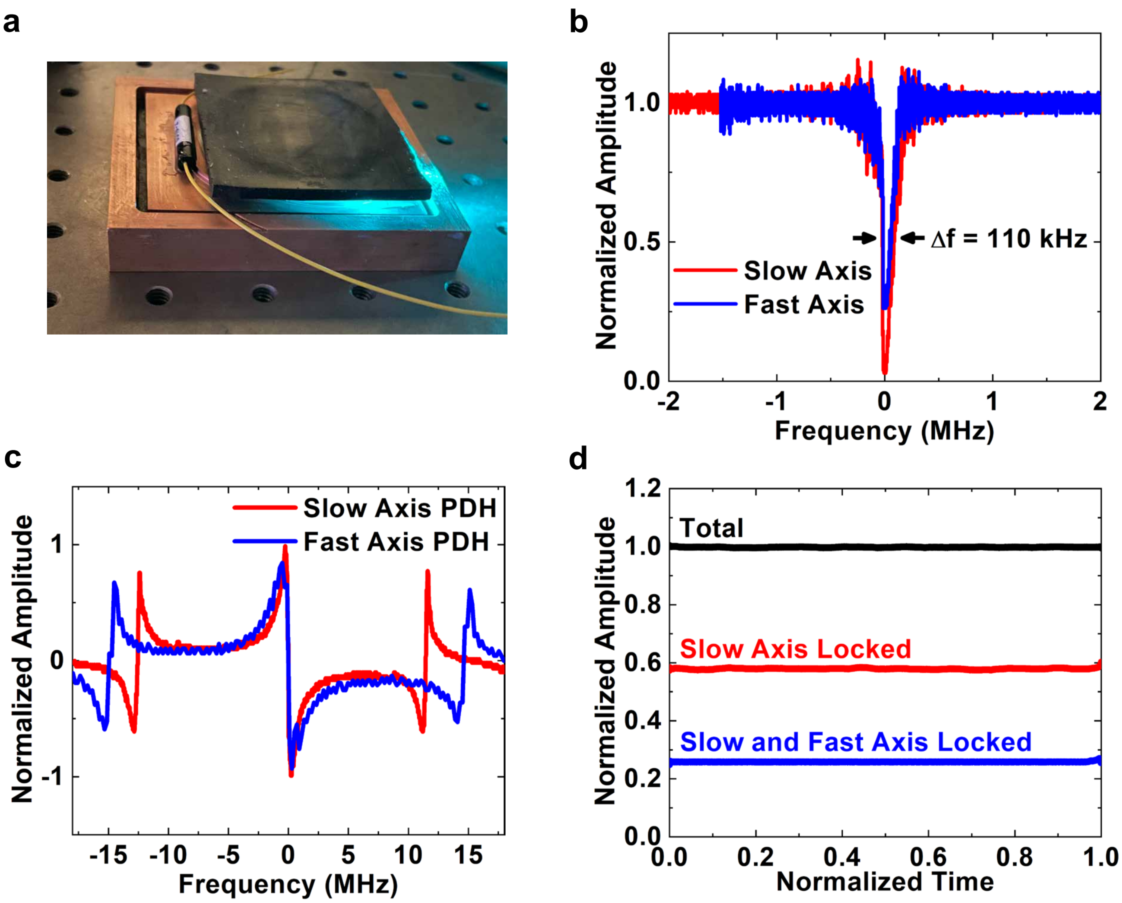

Supplementary Fig. 1. Stimulated Brillouin Scattering Resonator Operation a, 10-meter SBS fiber resting in a copper enclosure. The fiber is coiled on a copper cylinder and rests below a section of Viton that holds the fiber in place. **b**, Optical resonances of the slow axis and fast axis modes. The linewidth is measured to be 110 kHz. **c**, PDH error signals of the slow and fast axis modes. The sideband frequencies correspond to 12 MHz and 15 MHz for the slow and fast axis, respectively. **d**, Normalized amplitude of the photodetected light. After locking the slow axis, and subsequently the fast axis, the amplitude decreases to 25% of the total.

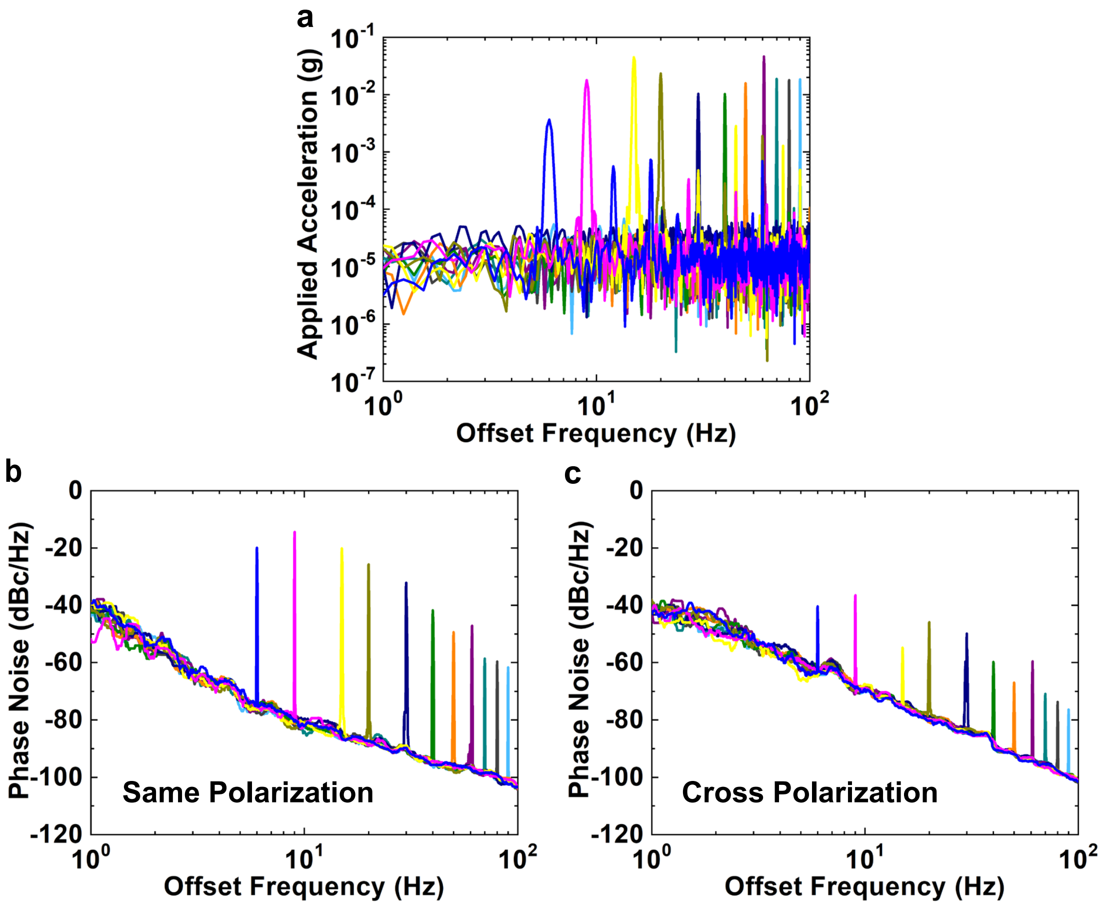

Supplementary Fig. 2. Optical Difference Frequency Division Vibration Response a, Applied platform acceleration along the Z-direction as measured by an accelerometer. The spectrum is measured with a resolution bandwidth of 1 Hz. **b**, Phase noise of ODFD microwave output in response to the applied acceleration. The system is operated with both SBS lasers locked along the same polarization. **c**, Phase noise of ODFD microwave output in response to the applied acceleration. The system is operated at the magic cancellation point with both SBS lasers locked along opposite polarization axes.
